# Towards Establishing a National Perioperative Quality Improvement Network in LMICs: Implementation Experiences From Ethiopia

**DOI:** 10.1097/AS9.0000000000000480

**Published:** 2024-08-19

**Authors:** Fitsum Kifle, Katherine R. Iverson, Ermiyas Belay, Elubabor Buno Teko, Abiy Dawit, Andualem Deneke, Bruce Biccard

**Affiliations:** * From the Network for Perioperative and Critical Care, Debre Berhan University Asrat Woldeyes Health Sciences Campus, Debre Berhan, Ethiopia; † Global Surgery Division, Department of Surgery, Faculty of Health Sciences, University of Cape Town, South Africa; ‡ Department of Surgery, Medical College of Wisconsin, Milwaukee, WI; § Department of Public Health, Wolkite University, Wolkite, Ethiopia; ∥ Medical Services, Ministry of Health, Addis Ababa, Ethiopia; ¶ Department of Surgery, School of Medicine, College of Health Sciences, Addis Ababa University, Addis Ababa, Ethiopia; # Department of Anaesthesia and Perioperative Medicine, Groote Schuur Hospital, University of Cape Town, Cape Town, Western Cape, South Africa.; Department of Surgery, Debre Berhan University, Asrat Woldeyes Health Sciences Campus; Department of Anaesthesia & Critical Care, Mbale Regional Referral Hospital, Mbale, Uganda; Department of Surgery, Debre Tabor University; Ministry of Health, Ethiopia; Department of Quality and Health Management Information System, Kidus Peteros Hospital, Addis Ababa, Ethiopia; Debre Berhan University, Asrat Woldeyes Health Sciences Campus; Department of Obstetrics and Gynaecology, Alert Hospital, Addis Ababa, Ethiopia; Department of Surgery, Medical College of Wisconsin, Milwaukee, WI; Department of Anesthesia, Wolkite University, Wolkite, Ethiopia; Department of Surgery, College of Medicine and Health Sciences, Arba Minch University, Ethiopia; Department of Anaesthesiology and Critical Care, Dr George Mukhari Academic Hospital, Sefako Makgatho Health Sciences University, Pretoria, South Africa; Department of Anaesthesia, Debre Berhan University, Asrat Woldeyes Health Sciences Campus; Department of Anaesthesia, Ayder Comprehensive Specialized Hospital, Mekelle, Tigray; Institute for Healthcare Improvement, Addis Ababa, Ethiopia; Medical Services, Ministry of Health, Addis Ababa, Ethiopia; Professor of Intensive Care Medicine, Queen Mary University of London, London, United Kingdom; Division of Global Surgery, Department of Surgery, University of Cape Town; Department of Anesthesiology and Critical Care, Kidus Peteros Hospital, Addis Ababa, Ethiopia; Department of Quality and Health Management Information System, Alert Hospital, Addis Ababa, Ethiopia

**Keywords:** perioperative, quality, data, Ethiopia

## CURRENT SCENARIO IN ETHIOPIA

Recently, Ethiopia has made significant improvements through government and international partnerships to advance perioperative care. The country has established a national surgical care strategic plan known as the Saving Lives Through Safe Surgery program and a guideline for perioperative patient management.^1,2^ Ethiopia is one of the few low- and middle-income countries that prioritize surgical care.^3,4^ Additionally, Ethiopia has expanded its surgical and anesthesia training programs and the number of facilities providing operative care.^5^ The Saving Lives Through Safe Surgery program has highlighted that the surgical procedures provided are short of the national target of 2500 per 100,000 population, that there is insufficient monitoring of the other specified targets (Table 1),^1,6^ that there is a lack of patient-level data to track patient progress and evaluate outcomes, and that there is limited interinstitutional collaboration hindering the road toward improved surgical care in Ethiopia. We established the National Perioperative Quality Improvement Network (NPQIN) to address these limitations in Ethiopia.

**TABLE 1.** Ethiopian National Surgical Plan 2025 Targets

Ethiopian MoH Targets for 2025
Reduce delay for elective surgery admission from 51 to 30 d
Conduct 2500 procedures per 100,000 populations by the end of 2025
Reduce the perioperative mortality rate to <2%
Achieve 100% tracking of surgical care–related deaths
Reduce the SSI rate to <5%
Increase the cesarean section rate from 4% to 10%
Provide 100% of woredas (districts) access to essential surgical care
Reduce anesthesia adverse events by 50%
Achieve 100% utilization of the SSC
Increase percentage of facilities providing basic surgical services from 44% to 80%
Reduce the number of clients on the waiting list for elective surgical service by 50%
Increase the proportion of health facilities with electricity from 76% to 100%
Increase the proportion of health facilities with an improved water supply from 59% to 90%

MoH indicates Ministry of Health; SSC, surgical safety checklist; SSI, surgical site infection.

### The Network’s Origin and Objectives

The Ethiopian NPQIN was founded by a team of clinicians and researchers with the objective of improving surgical quality and patient safety, in collaboration with network hospitals and the Ministry of Health. The network focuses on improving hospitals’ capacity to capture patient-level perioperative data, identifying areas of improvement, and implementing evidence-based interventions based on regular data analysis and visualization.

This partnership enables knowledge and experience sharing among perioperative care providers in hospitals, policymakers at the ministerial level, and clinical researchers. The goal is to ensure that best practices are implemented across all participating hospitals by providing regular virtual and in-person meetings (at least monthly, more frequently as needed), training sessions on updated guidelines and protocols (such as enhanced recovery after surgery and surgical site infection prevention), hosting workshops and conferences to discuss data and findings, and establishing a platform for continuous communication and feedback among all stakeholders involved. Furthermore, the network conducts regular audits and evaluations to monitor clinical practice and outcomes, identifies areas for improvement in perioperative care practices, and supports research collaboration.

**FIGURE 1.**
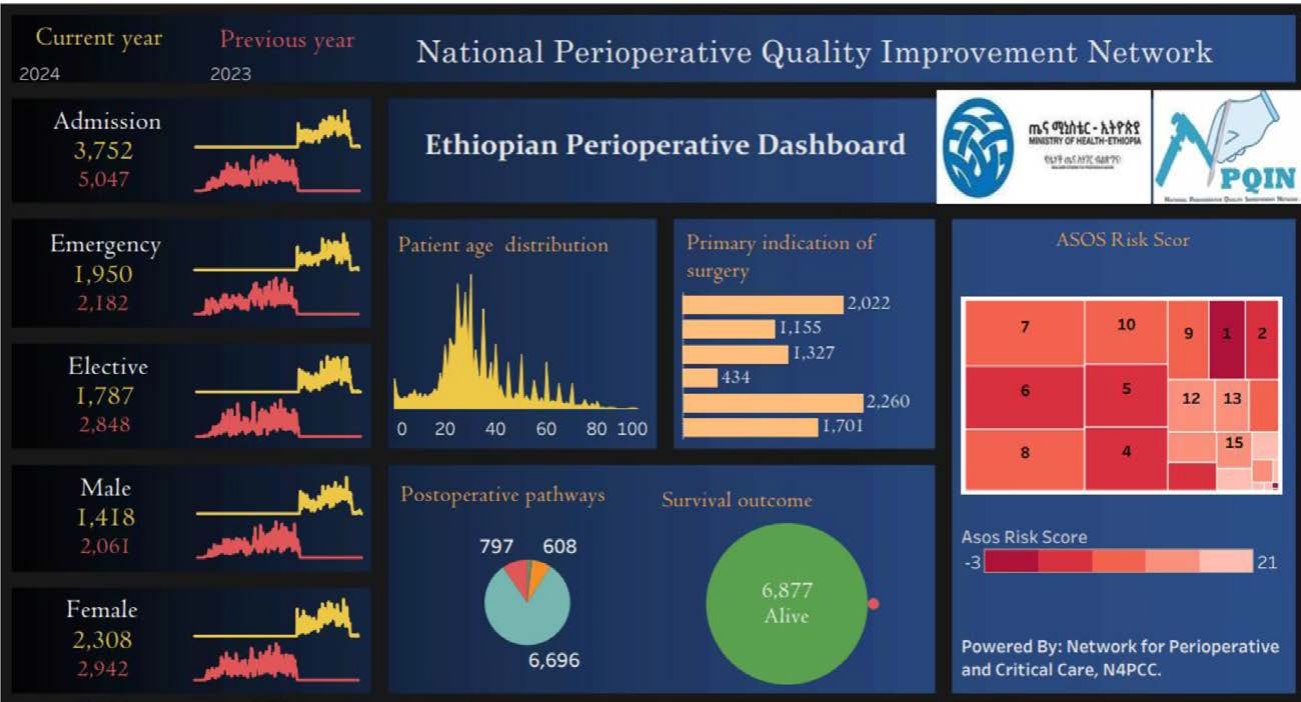
National Perioperative Quality Improvement Network (NPQIN) public dashboard (https://n4pcc.com/perioperative-registry-overview).

The NPQIN seeks to improve patient outcomes and the quality of perioperative care in Ethiopia by sharing aggregated and deidentified data needed to understand surgical complications, improve patient satisfaction, and optimize resource utilization. For example, NPQIN would track postoperative infection rates across sites and recommend evidence-based strategies, drawing from experience within the network and international guidelines. Collaboratively, the network engages hospitals in brainstorming intervention plans tailored to their contexts. Quality improvement programs from collaborating centers have resulted to address these issues. The network then assists in integrating the monitoring variables to evaluate the effectiveness of the planned intervention strategies. Through this approach, the network aims to promote continuous quality improvement and better patient outcomes. This is illustrated in the public dashboard (Fig. 1), which includes the Ministry of Health key indicators (eg, aggregated surgeries and associated mortalities), while the secured facility-level dashboards allow each hospital to add their own metrics. The registry tool and dashboard include the African Surgical Outcomes Study surgical risk calculator,^7^ which facilitates risk-adjusted outcomes and supports the introduction of evidence-based protocols for perioperative care. Integrating the African Surgical Outcomes Study surgical risk calculator facilitates risk-benefit preoperative consultation between patients and clinicians.

### Innovative Strategies and Collaborative Learning

The network’s collaborative model brings together a diverse group of hospitals, including primary, secondary, and tertiary institutions, spanning public, private, and nongovernmental organizations, fostering a comprehensive and holistic approach to improving perioperative care in Ethiopia. The foundational work for NPQN was piloting a cloud-hosted real-time perioperative registry on a small scale.^8^ It then expanded to multiple hospitals, improved data infrastructure, standardized data collection, and established management structures. This growth enabled NPQN to collect more comprehensive data, from preoperative admission to discharge information, providing deeper insights into perioperative care across different surgical facilities. NPQN now includes 10 hospitals in Ethiopia.

Monthly meetings are held to discuss surgical volume, data quality, and troubleshooting with site supervisors, hospital leads, and data collectors. The public dashboard (Fig. 1) is accessible to local and international academics, researchers, and the community for learning about surgery, developing surgical advocacy programs, and designing more research or improvement initiatives. Network hospitals have secure dashboards for local targeted analysis and decision-making. This has been accompanied with perioperative care training to address locally identified needs. Additionally, the network fosters knowledge sharing among hospitals, allowing for the dissemination of best practices and the implementation of evidence-based strategies.

### The Future Vision

The network aims to build upon the existing work to improve the quality of perioperative data using patient-level outcomes data. This includes capturing the 15 key performance indicators that are integrated into the hospital monitoring and evaluation system as part of the national surgical policy.^9^ Since the introduction of the Digital Health Information System nationally, these surgical metrics have been systematically collected and reported monthly for hospitals, reflecting a commitment to data-driven decision-making at a ministerial level. However, the creation of a perioperative registry has provided the opportunity to integrate patient-centric outcomes to further strengthen the hospital, regional, and national patient-level data necessary for quality improvement projects within the surgical service. The dataset also incorporated the African perioperative minimal dataset, as determined by the African Perioperative Research Group,^10^ which will enable comparisons and benchmarking of perioperative outcomes across different regions in Africa. This standardized dataset is expected to facilitate research collaboration and the development of evidence-based guidelines for perioperative care in Africa. Ultimately, this will allow for an international comparison of global surgery metrics, leading to improved patient care and surgical practice on the continent. The network has started to recruit additional facilities with a keen interest in tracking and improving perioperative outcomes and accepting requests to join the network. The number of hospitals in the network is expected to grow significantly in the future. Additionally, efforts are underway to collaborate with electronic medical record providers to integrate surgical variables into their systems, ensuring accurate and timely data collection across hospitals with and without electronic medical records systems.

In the future, we plan to develop community-based interventions in order to address the fear associated with publicly displaying surgery-related mortality data, which may result in patient anxiety for surgical procedures.^11^ We plan to improve patient communication with accessible information and support through patient education materials, brochures, live chatbots, and hotlines. Ongoing efforts are needed to standardize data collection for consistency and comparability among participating hospitals and their African counterparts with similar initiatives, enabling international outcome comparison. NPQN provides an example of how this can be achieved. Furthermore, the network intends to collaborate with international organizations and field experts to establish a robust network for knowledge sharing and capacity building. This includes designing and providing professional development courses with the goal of expanding the impact of the initiative on global surgical care.

## CONCLUSIONS

Collaboration and communication among stakeholders are crucial in resource-limited settings. Prioritizing data-driven decision-making can optimize outcomes, especially in low-resource environments. The establishment of NPQIN in Ethiopia serves as a test case for other countries. By sharing best practices and lessons learned, this collaborative approach not only improves patient safety and quality of care but also contributes to the overall healthcare system development.

## ACKNOWLEDGMENTS

NPQIN Collaboratives: Tewodros Kifleyohanes, Department of Surgery, Debre Berhan University, Asrat Woldeyes Health Sciences Campus; Adam Hewitt Smith, Department of Anaesthesia & Critical Care, Mbale Regional Referral Hospital, Mbale, Uganda; Atalel Fentahun, Department of Surgery, Debre Tabor University; Bereket Zelalem, Ministry of Health, Ethiopia; Bethel Muleye, Department of Quality and Health Management Information System, Kidus Peteros Hospital, Addis Ababa, Ethiopia; Bezaye Zemedkun, Debre Berhan University, Asrat Woldeyes Health Sciences Campus; Brook Demissie, Department of Obstetrics and Gynaecology, Alert Hospital, Addis Ababa, Ethiopia; Chris Dodgion, Department of Surgery, Medical College of Wisconsin, Milwaukee, WI; Dereje Zewdu, Department of Anesthesia, Wolkite University, Wolkite, Ethiopia; Desta Galcha, Department of Surgery, College of Medicine and Health Sciences, Arba Minch University, Ethiopia; Hyla Kluyts, Department of Anaesthesiology and Critical Care, Dr George Mukhari Academic Hospital, Sefako Makgatho Health Sciences University, Pretoria, South Africa; Kokeb Desta, Department of Anaesthesia, Debre Berhan University, Asrat Woldeyes Health Sciences Campus; Masersha G., Department of Anaesthesia, Ayder Comprehensive Specialized Hospital, Mekelle, Tigray; Mohammed Adem, Institute for Healthcare Improvement, Addis Ababa, Ethiopia; Natinael Tesfaye, Medical Services, Ministry of Health, Addis Ababa, Ethiopia; Prof Rupert M. Pearse MD (Res), Professor of Intensive Care Medicine, Queen Mary University of London, London, United Kingdom; Salome Maswime, Division of Global Surgery, Department of Surgery, University of Cape Town; Selam Daniel, Department of Anesthesiology and Critical Care, Kidus Peteros Hospital, Addis Ababa, Ethiopia; Yordanos Mengistu, Department of Quality and Health Management Information System, Alert Hospital, Addis Ababa, Ethiopia.
